# Co-occurrence, stability and manifestation of child and adolescent mental health problems: a latent transition analysis

**DOI:** 10.1186/s40359-022-00969-4

**Published:** 2022-11-14

**Authors:** Kristin Göbel, Niklas Ortelbach, Caroline Cohrdes, Franz Baumgarten, Ann-Katrin Meyrose, Ulrike Ravens-Sieberer, Herbert Scheithauer

**Affiliations:** 1 Mental Health Research Unit, Department of Epidemiology and Health Monitoring, Robert Koch Institute, Berlin, Germany; 2 Division of Developmental Science and Applied Developmental Psychology, Department of Education and Psychology, Freie Universität Berlin, Berlin, Germany; 3 Child Public Health, Department of Child and Adolescent Psychiatry, Psychotherapy, and Psychosomatics, University Medical Center Hamburg-Eppendorf, Hamburg, Germany; 4 Clinical Psychology, Helmut Schmidt University/University of the Federal Armed Forces Hamburg, Hamburg, Germany

**Keywords:** SDQ, Mental health problems, LTA, Psychiatric disorders, Childhood

## Abstract

**Background:**

Complex constellations of socio-emotional and behavioural problems (i.e., mental health problems) in childhood and adolescence are common and heighten the risk for subsequent personality, anxiety and mood disorders in adulthood. Aims of this study included the examination of patterns of mental health problems (e.g., externalizing-internalizing co-occurrence) and their transitions to reported mental disorders by using a longitudinal person-centered approach (latent class and latent transition analysis).

**Methods:**

The sample consisted of 1255 children and adolescents (51.7% female, mean age = 12.3 years, age range 8–26 years) from three time points of the comprehensive mental health and wellbeing BELLA study. Children and their parents completed the German SDQ (Strength and Difficulties Questionnaire, Goodman, 1997) and reported on diagnoses of ADHD, depression, and anxiety.

**Results:**

Latent class analysis identified a normative class, an emotional problem class, and a multiple problem class. According to latent transition analysis, the majority of the sample (91.6%) did not change latent class membership over time; 14.7% of individuals showed a persistent pattern of mental health problems. Diagnoses of mental disorders were more likely to be reported by individuals in the emotional problem or multiple problem class.

**Conclusions:**

Results highlight the need for early prevention of mental health problems to avoid accumulation and manifestation in the transition to adolescence and young adulthood.

## Introduction

Worldwide, 10 to 20% in children and adolescents are affected by socio-emotional and behavioural adjustment problems starting early as infancy [1–4]. Substantial research has shown that multiple risk factors portend maladjustment in children and adolescents [5–7]. For instance, family dysfunction, parental psychopathology, low socioeconomic status, and family instability are strong indicators associated with mental health problems [8, 9]. Moreover, the highly sensitive developmental period from infancy to late adolescence also entails multiple biological (e.g., puberty), cognitive (e.g., identity formation), and socio-environmental (e.g., school transition) changes [10], making the development of mental health problems more likely if adjustment to these challenges fails. Beyond that, mental health problems in childhood and adolescence can exert a long-term impact on adult life, family and society. Important sequelae include the risk of later delinquency, substance abuse, maladaptive social relationships, loss of productivity, work incapacity, and increased costs in health care and education [11, 12].

The high prevalence, the early onset, the related functional impairment, and the subsequent societal burden acknowledge mental health problems as a major public health challenge [1, 11, 13, 14]. Thus, due to the growing burden of mental ill-health, it is of great importance to identify complex constellation of socio-emotional and behavioural problems, their stability, and manifestation in children and adolescents at an early stage to develop effective intervention strategies in order to promote healthy development.

## Co-occurrence of internalizing and externalizing problems

Mental health problems or psychopathological symptoms are commonly conceptualized in two broad dimensions of internalizing and externalizing problems [8, 15–17]. While internalizing problems are described as social-emotional problems directed inwards and related to social withdrawal, depression and anxiety, inversely externalizing problems are behavioural problems directed outwards such as hyperactivity, non-compliance, aggression [18]. Externalizing problems are consistently found to be more common in early adolescent boys, in turn internalizing problems are more prevalent in late adolescent girls [8].

Beyond that, the strict separation of mental health problems into two dimensions as behavioural (i.e., externalizing) and socio-emotional (i.e., internalizing) problems may be an over-simplification as they are not mutually exclusive [19]. Although the literature predominantly consists of studies focusing on internalizing or externalizing problems only, research indicates the co-occurrence of socio-emotional and behavioural problems [19–23]. Several studies showed a close interplay between internalizing and externalizing problems providing evidence for the existence of a separate “co-occurrence” dimension by which children and adolescents experience both problems at the same time [8, 15, 16, 19, 24–27]. Research that examined internalizing-externalizing co-occurrence reported varying prevalence rates with the smallest rate for preschool children (2%) [8, 16], while higher rates were identified during middle childhood (5–10%) [24, 25], and adolescence with 20% reporting co-occurring problems [19]. Externalizing-internalizing co-occurrence, compared to sole mental health problems, is associated with a greater severity of symptoms, a higher persistence into adulthood and is linked to more adverse outcomes [8, 16, 28].

Mental health problems are often regarded as transient meaning that problems tend to diminish with growing age due to the rapid developmental changes in childhood [15]. Conflicting with this view, a vast amount of evidence suggests a persistence of early internalizing and externalizing problems from childhood into adolescence and adulthood [10, 16, 21, 29–31]. Becker et al. [31] examined children and adolescents (6–18 years) from Germany using the Strengths and Difficulties Questionnaire SDQ [32]; and showed that 14 to 21% of children and adolescents with abnormal scores on the SDQ-scales at baseline remained “abnormal” over a time period of 6 years. Several findings support previous indications of the continuity of the same problems over time (i.e., homotypic stability) and/or cross-dimensional effects (i.e., heterotypic stability) [26, 27, 33, 34]. The continuity of high levels of childhood socio-emotional and behavioural problems are related to diagnoses of mental disorders in adulthood, although especially co-occurring problems have been identified to be persistent [14, 35]. Picoito and colleagues [26] found a persistent mixed externalizing and internalizing profile at age 5 continuing into adolescence. The persistence of co-occurring problems is stronger compared to either problem alone, especially for young children [4, 8, 15, 16]. In addition to the higher persistence of co-occurring externalizing and internalizing problems, it is well known that co-development is associated with more severe adverse outcomes (e.g., mental disorders, substance abuse, deviant behaviour, risk for suicide) [8, 23, 36, 37].

Based on the gravity of co-occurring problems, an increasing demand for intervention programmes directed towards both types of problems (i.e., internalizing and externalizing) has been claimed rather than targeting either problem alone [38–40].

General scientific research on mental health problems among children and adolescence has largely relied on variable-centered methodology (e.g., multiple regression, factor analysis) examining internalizing and externalizing problems [10, 41, 42]. Despite its usefulness, the method assumes homogeneity of mental health problems within a population, however there is likelihood for diversity of individual patterns for types of problems [43, 44]. Meaning that some subgroups of children or adolescents remain in the same group over time while others shift to more complex (or simpler) presentations of symptoms in different groups. An appropriate and promising method to identify individual patterns of psychopathology and its stability over time is the use of a longitudinal person-centered technique.

## Latent transition analysis

An empirical approach to investigate complex combinations of problem behaviours is a person-centered technique such as Latent class analysis (LCA) or latent transition analysis (LTA) [45]. LTA is a longitudinal extension to the LCA, which identifies homogeneous patterns or profiles of individuals cross-sectional according to their response pattern on a given number of categorical variables [46]. LTA examines changes in the combination of those patterns over time or age also called latent transitions. Relative to a variable-centered approach (e.g., confirmatory factor analysis), those person-centered methods have the advantage that they assume a heterogeneity within the population regarding the influence of predictors. Previous research on mental health problems among children and adolescents has tended to provide data based on variable-centered methods that capture valuable information about relations between variables (e.g. [47]). Despite its usefulness, they lack the ability to reveal the diversity of individual patterns. In contrast, LCA captures patterns of characteristics in subgroups. Furthermore, LCA classes are not based on cut-points but derived empirically. The person-centered approach may not substitute variable-centered techniques but rather give more insight into typical patterns of mental health problems [48].

In recent years, with the growing popularity of person-centered approach, several studies used LCA to determine patterns of mental health problems in subgroups [19, 25, 49–51]. Olino and colleagues [49] examined patterns of lifetime internalizing and externalizing psychopathology using LCA on an adolescent US sample. A four-class solution identified a ‘normative’ subgroup (62.5%); an ‘internalizing disorders’ subgroup (16.4%); an ‘externalizing disorders’ subgroup (16.9%); and the fourth class was characterized by both internalizing and externalizing disorders (4.2%). A more recent study by Ling and colleagues [19] published three distinct subtypes of internalizing and externalizing behaviours in Chinese adolescents using LCA method—a high-risk group, a middle-risk group, and a low-risk group. The main finding suggested that Chinese adolescents showing elevated mental health problems in one domain also exhibited problems in the other domain.

Relatively few studies have used a LTA to examine typical patterns of co-occurring mental health symptoms and transition between patterns over time during childhood and adolescence [26, 45]. McElroy et al. [45] conducted a LTA to model comorbidity of eight DSM-IV disorders from childhood (age 7.5 years) to adolescence (age 14 years) [45]. The 4-class solution identified a normative class, an internalizing class, an externalizing class, and a high risk/multimorbid class. Results suggested a very high stability of latent classes over time (80%). Additionally, Picoito and colleagues [26] used a LTA to show continuity of mental health problems across different developmental stages—greater heterotypic stability between ages 3 and 5 (transition from high to moderate profiles), while homotypic transitions are more likely for children between 5 and 11 years with a consistent high probability to remain in the same profile.

Lanza [52] described in her dissertation children (preschool, Grade 1, 3, 4, 5, 6) characterized by three latent classes based on oppositional defiant disorder (ODD) and depressive symptom severity. The first group showed very low levels of ODD or depressive symptoms, a second ODD-only group (with low levels of symptoms), and a third co-occurring ODD and depressive symptom group with moderate levels of ODD and low levels of depressive symptoms.

Although some research has been done person-oriented methods to capture information on the individual level to distinguish patterns of characteristics across subgroups. Only little research examined externalizing-internalizing co-occurrence, the transitions of patterns over time and to our knowledge no study has investigated its association with psychiatric disorders in the transition to adulthood, yet. Identification of complex mental health patterns and their manifestation into adult psychiatric disorders would help to gain more insight into early structure and development of psychiatric disorder and therefore crucial for tailored intervention tools in childhood and adolescence.

## The current study

The current study extends upon previous research by examining patterns of mental health problems, its co-occurrence, stability over time, and its manifestation into self-reported diagnosis of psychiatric disorders using a longitudinal person-centered approach (LCA and LTA). The first aim was to investigate the structure and prevalence of profiles of mental health problems among children and adolescents (8–18 years) using the SDQ sub-scales hyperactivity/inattention, emotional, conduct, and peer relationship problems [17]. As binary or categorical scoring of the SDQ is very often used by researchers to reveal the presence or absence of mental health problems, a latent class analysis was chosen as the primary analysis model [23, 53, 54].

In contrast to the focus on preschool or adult samples as in most previous studies (e.g., [16, 55, 56]), this research uses a wide age range covering symptoms during childhood, adolescence, and early adulthood. Additionally, earlier studies used either cross-sectional data to identify subgroups of mental health problems [19] or descriptive, variable-centered approaches to investigate trajectories of mental health problems [31, 57]. However, those results capture only "single problems" and do not consider individual patterns of mental health problems and transitions over time. Our study addresses this issue using a longitudinal person-centered approach (i.e., LTA). Furthermore, to our knowledge only two studies have examined the internationally validated screening instrument Strength and Difficulties Questionnaire (SDQ) to explore characteristics in subgroups so far (see [26, 58]).

Finally, contrary to previous research using the SDQ, not only parental reports were analyzed but a multi-informant measure of mental health problems including parent- as well as self-reports are applied as best-practice approach to assess mental health problems [59].

Based on previous findings using a person-centered approach to empirically derive patterns of mental health problems (e.g., [26, 45, 52]), we primarily expect the emergence of a four class solution that broadly reflects: internalizing problems, externalizing problems, a class characterized by no or low mental health problems, and a class characterized by co-occurring problems. The second aim was to examine the stability of these patterns of mental health problems over three time points covering the time span of 5 years using LTA. According to recent literature investigating LTA patterns of mental health [26, 45], we expect that children with co-occurring problems are more likely to show persisting symptoms.

Lastly, to determine associations of distinct mental health problem profiles with self-reported diagnoses of psychiatric disorders (e.g., ADHD, depression, and anxiety), several mental health outcomes were included in the analyses. To our knowledge, no previous person-centered study has examined individual patterns over time (i.e., stability vs. variability) and their association with reported psychiatric disorders in transition to late adolescence and early adulthood. Based on empirical literature, we expect a stronger association of co-occurrence of internalizing and externalizing behaviour problems with self-reported diagnosis of psychiatric disorders compared to single mental health problems [14, 35].

## Method

### Sample

The current study utilized data from the comprehensive BELLA study, a module on mental health and wellbeing of the German National Health Interview and Examination Survey for children and adolescents (KiGGS). The BELLA study assessed 2863 children and adolescents aged 7–17 years and their parents at baseline from 2003 to 2006 with three follow-ups after 1 year (B1 = 2004–2007), 2 years (B2 = 2005–2008) and 6 years (B3 = 2009–2012). A more detailed description regarding conceptualization and design is provided by Ravens-Sieberer and colleagues [60, 61].

The present analyses focused on 1255 children and adolescents from three follow-ups of the BELLA study (B1, B2, and B3). The gender distribution was 51.7% female and the age range covered participants from 8 to 26 years with a mean age of 12.3 years (B1, SD = 3.11), 13.3 years (B2, SD = 3.11) and 18.5 years (B3, SD = 3.24). Logistic regression analysis predicting missing data at a specific time point indicated that participants with a migration background (OR 1.64, 95% CI 1.01–2.67) and female participants at B3 (OR 1.85, 95% CI 1.17–2.93) were significantly more likely to show missing values for mental health problems compared to non-migrants and males. SES showed no significant association with missings. The 2-year follow-up (B2) had no missing answers. Additional details on study design, sampling, and attrition can be found in Ravens-Sieberer and colleagues [62]. Participants with available data on mental health problems for at least two follow-ups (i.e., B1 and B2) and outcome variables (at B3) such as self-reported diagnoses of depression, ADHD, and anxiety were included in the analyses.

### Measures

Mental health problems. Children and their parents completed the German SDQ Strength and Difficulties Questionnaire, [32] at each time point (i.e., B1, B2, and B3), which is a validated questionnaire with 20 items screening mental health problems on the subscales hyperactivity/inattention, emotional, conduct, and peer relationship problems, which are also used equivalent to internalizing and externalizing problems [63, 64]. Items are answered based on a Likert-type scale ranging from ‘‘not true’’, ‘‘somewhat true’’ to ‘‘certainly true’’. The SDQ is widely used as a screening instrument for mental health problems in children and adolescents, in addition it has also been described as a sensitive measure of treatment outcomes closely predicting the proportion of children with a mental disorder [41]. A score for each subscale was summed and banding scores were used to categorize participant’s mental health problems into ‘‘abnormal’’, ‘‘borderline’’, or ‘‘normal’’ (see [65, 66] for further information). A conservative approach was adopted to transform scores of each subscale into a binary variable by combining “borderline” and “normal” scores. A binary variable (i.e., “abnormal” vs “normal and borderline”) facilitates the interpretation of latent classes and reduces the number of parameters estimated by the models [67]. The present study used the four SDQ subscales to determine patterns of mental health problems, excluding the fifth scale "prosocial behaviour". The SDQ parent and self-report were administered at each time point (i.e., B1, B2 and B3). The self-report was used for children older than 11, while the parent-report was considered from age 7.

To increase the accuracy of diagnostic predictions generated by the SDQ, we included a multiple informant approach using self- and parent-reports. Information drawn from multiple informants is more valuable as for instance, in case of emotional problems teenagers may report worries or fears that they have hidden from parents. The self- and parent-reported scores were aggregated based on the predictive algorithm published by Goodman et al. [68]. Briefly, self-reported scores were prioritized over parent-reports for the subscales “peer problems” and “conduct problems”. Self-reports for “hyperactivity/inattention” were only considered in case of missing parental reports, and finally, self- and parent-reports were both considered to determine “emotional problems”. The SDQ has demonstrated sound psychometric properties [59, 69, 70], is used in clinical practice and published in numerous studies [58, 71]. Moreover, measurement invariance for the SDQ was shown by Murray et al. [72] suggesting comparability across developmental trajectories from early childhood into adolescence and across gender.

Mental health outcomes. At the last time point (i.e., B3), participants reported if they were ever diagnosed with a mental disorder according to ICD-10 criteria by either a physician or a psychologist. Diagnoses of attention deficit hyperactivity disorder (ADHD), depression, and anxiety coded as “yes” or “no” are used as outcome for the LTA model.

### Data analysis

All analytical steps were estimated with the software Mplus Version 7.4 (https://www.statmodel.com/) and Stata SE version 14 (http://www.stata.com/). Mplus incorporates all cases under the missing at random (MAR) assumption using the full information maximum likelihood approach.

The first aim of this study included the examination of profiles of mental health problems across a sample of children and adolescents for each time point (using LCA). The second and third aim included the investigation of transition of subgroups of mental health problems over time points (i.e., stability) into reported mental disorders (i.e., manifestation). More precisely, LTA includes a measurement model which describes the structure of latent classes at different time points, while the autoregressive model examines individual movement or transition between classes over time [67].

LTA model was specified using the following model-building steps as suggested by Nylund [67]. In a first step, successive LCA models with ascending number of classes were specified and tested separately for each time point using the four binary mental health indicators (emotional, peer, hyperactivity, and conduct problems) and covariates (gender, age) using the R3STEP method in Mplus [73]. The appropriate number of latent classes was determined using several fit statistics including Akaike information criterion (AIC), Bayesian information criterion (BIC), and the sample size-adjusted Bayesian criterion (aBIC), with lower values indicating better fit. The Lo–Mendel–Rubin likelihood ratio test (LMR-LRT) and Bootstrap Likelihood Ratio Test (BLRT) were used to compare (K-1)-class models to a K-class model. A significant *p* value (*p* < 0.05) was indicative of a better model fit for the respective K-class model. In addition, the entropy and posterior probabilities were examined for each model. After the number of classes had been selected, measurement invariance was investigated in a second step, to determine whether the measurement model remained consistent over time. Thereby, it was investigated if class solution at B1 has the same structure and meaning as class solution at B2 and B3. Competing models with different levels of conditional item probability constraints were tested for stability over time. Two different levels of measurement invariance were explored: full measurement invariance (i.e., conditional item probabilities constrained to be equal at all time points) and measurement non-invariance (i.e., conditional item probabilities freely estimated over time). Models were compared using the Log Likelihood Ratio Test (LRT) and model fit indices. Third, a LTA model with relevant covariates using the 3-step procedure was calculated such as participants’ gender and age to ensure classification accuracy of our model [74, 75]. Furthermore, individuals were classified as ‘movers’ (i.e., those who transition to a different class over time) or ‘stayers’ (i.e., those who remain in the same class over time) to study the stability and change of class membership over time. Finally, our analysis included a set of mental health outcomes (i.e., self-reported diagnoses of depression, ADHD and anxiety) to study their association with latent class membership and latent transition patterns.

## Results

### Descriptive statistics

The proportion of children endorsing each SDQ subscale at each time point is reported in Table 1. Emotional problems were reported most commonly, followed by peer and conduct problems, and hyperactivity was reported least frequently. A small non-significant increase for emotional problems was visible between time point B1 and B3. Multinomial regression analyses were performed to test for significant differences between time points. Results revealed a significant decrease of hyperactivity values attime point B3 compared to B2 (RRR = 0.42, 95% CI 0.25–0.72) and B1 (RRR = 0.33, 95% CI 0.19–0.57).

**Table 1:** Proportions and 95% confidence intervals of participants reporting specific mental health problems as indicated by the SDQ (normal/borderline vs. abnormal), grouped by time point (N = 1255)

SDQ-subscales	B1 (95% CI)	B2 (95% CI)	B3 (95% CI)
Emotional problems	11.61 (10.08–13.80)	12.91 (11.24–14.97)	13.88 (11.71–16.89)
Peer problems	9.81 (8.34–11.79)	10.55 (8.93–12.34)	9.84 (7.70–12.11)
Hyperactivity	6.70 (5.53–8.45)	5.51 (4.43–6.99)	2.43^a^ (1.51–3.86)
Conduct problems	9.73 (8.03–11.42)	9.37 (7.91–11.15)	10.78 (8.34–12.89)

B1 = 2004–2007, B2 = 2005–2008, B3 = 2009–2012

^a^Significantly decreases compared to B1 and B2

### Latent class and latent transition analysis

*Step 1* Best measurement model

Following a series of latent class analyses for each time point (B1, B2, and B3) with covariates and their fit statistics, a three-class solution was identified as optimal. Entropy values were of similar magnitude for the three-class solution over all time points. Table 2 presents the fit statistics for the different class solutions at each time point. LMR-LRT and BLRT values turned non-significant when four or five class solutions were specified for each time point. The number of classes is decided upon fit statistics, theory and interpretability [76]. As such, based on the fit statistics, interpretability and overall model parsimony, the three-class solution represents the best fitting measurement model at all time points and will be used for further analyses.

**Table 2:** Fit statistics for 2- to 5-class solutions as derived from LCA at time points B1, B2 and B3

Model	AIC	BIC	aBIC	Entropy	LMRT *p* value	BLRT *p* value
B1						
2-class	5.691.412	5.742.812	5.714.217	0.699	0.000	0.000
**3-class**	**5.675.873**	**5.755.828**	**5.711.348**	**0.862**	**0.000**	**0.000**
4-class	5.684.131	5.792.642	5.732.276	0.799	0.388	0.474
5-class	5.694.131	5.831.197	5.754.946	0.671	0.232	1.000
B2						
2-class	5.488.616	5.539.821	5.511.226	0.681	0.000	0.000
**3-class**	**5.476.521**	**5.556.172**	**5.511.692**	**0.814**	**0.000**	**0.000**
4-class	5.486.494	5.594.592	5.534.226	0.700	0.545	0.978
5-class	5.496.494	5.633.039	5.556.787	0.467	0.739	1.000
B3						
2-class	7.097.444	7.150.918	7.122.322	0.719	0.000	0.000
**3-class**	**7.077.793**	**7.160.976**	**7.116.493**	**0.855**	**0.002**	**0.000**
4-class	7.083.146	7.196.038	7.135.668	0.844	0.082	0.086
5-class	7.093.146	7.235.746	7.159.490	0.579	1.000	1.000

B1 = 2004–2007, B2 = 2005–2008, B3 = 2009–2012; AIC, Akaike information criteria; BIC, Bayesian information criteria; aBIC, adjusted BIC; LMR LTR *p* value, Lo–Mendell–Rubin likelihood ratio test *p* value; BLRT LRT p, Bootstrapped Likelihood ratio test *p* value; Best-fitting models by time points are indicated in bold

*Step 2* Measurement invariance

As confirmed by the LCA, the same number of classes were present over the three time points leading to the assumption of measurement invariance. Measurement invariance was examined by using the Log Likelihood Ratio Test (LRT) to compare the unconditional model (full measurement non-invariance) and the model with constrains (full measurement invariance). The LRT indicated a significant difference in model fit when constrains were added (Δχ2 = -44.4, *df* = 24, *p* < 0.05). Inspection of the BIC and adjusted BIC suggested a better model fit for the full measurement invariance model (BIC = 7638.902; aBIC = 7581.724) compared to the non-invariance model (BIC = 7777.670; aBIC = 7644.255). Full measurement invariance was supported, suggesting that, the structure of mental health problems was distributed in a similar manner across time points. Thus, full measurement invariance was used in the following analyses.

*Step 3* Latent transition analysis with covariates.

**Fig. 1:**
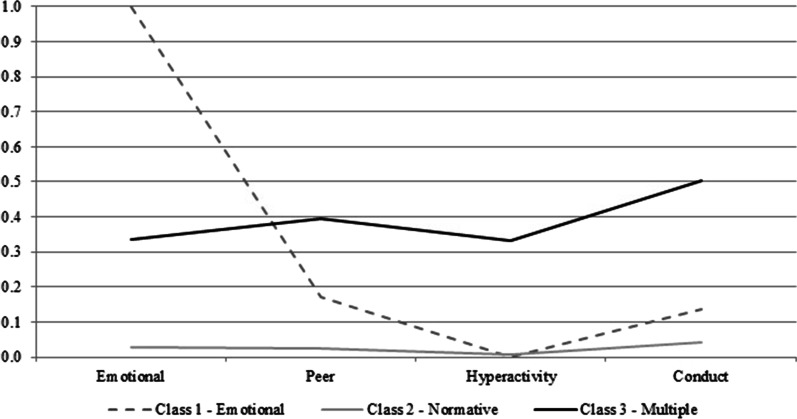
Conditional item probability plots in a model assuming measurement invariance

The item probability plot for the conditional model is shown in Fig. 1. The horizontal axis displays the four SDQ-subscales indicating mental health problems, while the vertical axis shows item probabilities. As predicted by the cross-sectional analyses, the three class solution at all time points contained a normative class (82.5% at B1; 80.7% at B2; 80.5% at B3) with a very low probability to show any mental health problem; an emotional problem class (3.9% at B1; 6.2% at B2; 8.2% at B3) with a very high probability to show emotional problems, low probability of conduct and peer problems, non-occurrence of hyperactivity; and a multiple problem class (13.6% at B1; 13.1% at B2; 11.3% at B3) with a medium probability to endorse more than one problem (i.e., hyperactivity, conduct, peer and emotional problems).

The proportion of the emotional problem class increased significantly over time.

In general, females were underrepresented in the normative class (RRR = 0.08, *p* < 0.001) and the multiple problem class (RRR = 0.05, *p* < 0.05) but overrepresented in the emotional problem class compared to males. The proportion of younger individuals was higher in the multiple problem class compared to the emotional problem class (RRR = 1.08, *p* < 0.001) and normative class (RRR = 1.07, *p* = 0.001).

Conditional transition patterns are displayed in Table 3. The transition probabilities represent the likelihood to move between classes over time points (i.e., B1, B2, and B3). Precisely, stability of class membership was very high from B1 to B2 for the normative class (85.5%), the emotional problem (96.8%) and the multiple problem class (90.9%). The transition from B2 to B3 was stable for the normative class (92.7%) and relatively constant for the multiple problem class (83%). In contrast, 63.2% of participants in the emotional problem class at B2 persisted in the class at B3 with 31.6% participants shifting from the emotional problem class to the normative class.

**Table 3:** Latent transition probabilities of the 3-class solution based on a conditional model with covariates comparing the time points B1 vs. B2 and B2 vs. B3

B1	B2		
	Emotional (6.2%)	Normative (80.7%)	Multiple problem (13.1%)
Emotional (3.9%)	0.855	0.145	0.000
Normative (82.5%)	0.032	0.968	0.000
Multiple problem (13.6%)	0.000	0.091	0.909

B2	B3		
	Emotional (8.2%)	Normative (80.5%)	Multiple problem (11.3%)
Emotional	0.632	0.316	0.052
Normative	0.073	0.927	0.000
Multiple problem	0.043	0.127	0.830

Each row represents the probability of individuals starting out in a given class (time point B1) transitioning to another class across B2 and B3. The diagonal probabilities represent the stability of class membership across time points

* Step 4* LTA transition patterns and mental health outcomes

Table 4 shows the prevalence of each class combination over time (i.e., LTA transition patterns and movers/stayers). The majority of the sample was classified as stayers (91.6%). More precisely, 11.3% of all individuals starting out in the multiple problem class remained in this class about 5 years later (at B3), while 2.9% of children in the sample showed an emotional problem pattern over three time points with a higher likelihood for females compared to males (*p* < 0.001). 77.3% of children and adolescents reported throughout a low level of mental health problems.

**Table 4:** Number of individuals and prevalence of latent transition patterns differentiated between movers and stayers (N = 1.255)

Time point	B1	B2	B3	n	% within movers/stayers	% of total sample
Stayers (91.6%)	Normative	Normative	Normative	970	84.4	77.3
	Multiple	Multiple	Multiple	142	12.4	11.3
	Emotional	Emotional	Emotional	37	3.2	2.9
Movers (8.4%)	Normative	Normative	Emotional	35	33.0	2.8
	Normative	Emotional	Emotional	25	23.6	1.9
	Multiple	Multiple	Normative	16	15.1	1.3
	Emotional	Emotional	Normative	11	10.4	0.9
	Multiple	Normative	Normative	7	6.6	0.6
	Multiple	Multiple	Emotional	6	5.7	0.5
	Normative	Emotional	Normative	5	4.7	0.4
	Emotional	Normative	Normative	1	0.9	0.2

With regard to movers, 8.4% of individuals showed a mental health problem pattern over time characterized by movement between different latent classes. Individuals with a mover pattern were significantly younger compared to stayers (16.9 vs. 18.6 years). Overall, eleven unique patterns emerged. The majority of patterns included the transition from the normative class to the emotional problem class (23.6%, 33.0% within movers). Albeit patterns of transition from the multiple problem class to the normative or emotional problem class were identified relatively often (Table 4), the reverse pattern from the normative or emotional problem class to the multiple problem class was nonexistent in the sample.

Conclusively, 14.7% of individuals in the sample showed a persistent pattern of mental health problems (multiple, emotional or both) over time at B1, B2, and B3.

Several mental health outcomes assessed at B3 and their association with class membership and LTA transition patterns were tested. Diagnoses of depression and ADHD were reported in the sample with 5.3% and 5.2%, respectively. While lower rates were found for diagnoses of anxiety disorders (2.6%). Table 5 shows the results for mental health outcomes and class membership at B3.

**Table 5:** Association between class membership and self-reported diagnoses of psychiatric disorders

Latent classes	Depression Adjusted RRR (95% CI)	Anxiety Adjusted RRR (95% CI)	ADHD Adjusted RRR (95% CI)
Emotional problem vs. Normative Class	3.96* (1.23, 12.70)	6.76* (1.34, 34.01)	3.71** (1.22, 11.21)
Multiple problem vs. Normative Class	5.21** (1.94, 13.90)	5.11* (1.20, 21.74)	9.03*** (5.14, 15.86)
Emotional problem vs. Multiple problem Class	1.31 (0.32, 5.23)	0.76 (0.11, 5.02)	2.44 (0.78, 7.54)

Results adjusted for gender and age; RRR relative-risk ratio; CI confidence intervals

*p < 0.05; **p < 0.01; **p < 0.001

Self-reported diagnoses of psychiatric disorders (i.e., depression, anxiety, and ADHD) were more likely to be reported by individuals in the emotional problem or multiple problem class compared to the normative class.

Multinomial regression analysis for transition patterns showed an increased likelihood for self-reported diagnoses of mental disorders for individuals with stable mental health problems. After controlling for age and gender, adolescents with a stable transition pattern for the emotional problem class are more likely to report diagnoses of anxiety disorders (RRR = 2.59, *p* = 0.003) and depression (RRR = 1.83, *p* = 0.006) compared to individuals without mental health problems across all three time points. Equally, individuals with a stable membership in the multiple problem class showed an increased likelihood to report a diagnosis of ADHD (RRR = 2.35, *p* < 0.001), depression (RRR = 1.85, *p* < 0.001) and anxiety disorder (RRR = 1.86, *p* = 0.015) compared to individuals without mental health problems over time.

In contrast, only one “mover” transition pattern showed a likelihood for reporting psychiatric disorders. After controlling for age and gender, individuals who transition from the multiple problem class (B1 and B2) to the normative class (RRR = 2.64, *p* < 0.001) at time point B3 were more likely to have reported a diagnosis of ADHD.

## Discussion

Aims of the present study included the examination of subgroups of mental health problems (e.g., internalizing-externalizing co-occurrence) across a sample of children and adolescents (using latent class analysis), as well as the investigation of transition of subgroups of mental health problems across three time points (i.e., stability) into self-reported psychiatric disorders (i.e., manifestation). A three-class solution was identified with a normative class, an emotional problem class, and a multiple problem class. The majority of the sample (91.6%) did not change latent class membership over time; 14.7% of individuals showed a persistent pattern of mental health problems. Diagnoses of mental disorders were more likely to be reported by individuals in the emotional or multiple problem class.

The present study is the first to apply LTA methodology to identify patterns of mental health problems over time using the multi-informant SDQ and to investigate later outcomes of mental disorders (self-reported diagnoses of depression, anxiety, ADHD).

### Class membership

Using LTA, the present study identified heterogenous subgroups in a German sample of children and adolescents over three time points (a period of five years). The three-class solution was selected and supported by full measurement invariance as appropriate across all three time points. As expected, the largest extracted class represented a group of children and adolescents with a low level of mental health problems. As the transition from childhood to adolescence is a vulnerable period with multiple biological and social changes (e.g., puberty, school transition), the presence of mental health problems might be within limits—without clinical relevance—a common part of variation in normal development [8, 10, 45].

Against our expectation to identify four latent classes for mental health problems, no class characterized solely by externalizing problems (i.e., hyperactivity and conduct problems) has been extracted from the data. Although the reason for not identifying an externalizing class is unclear, there are few possible explanations. It is notable that our sample does not include the period of early childhood. As externalizing problems decline or even diminish with increasing age and maturation due to the development of cognition and emotion regulation [8, 77, 78], it may suggest an insufficient large sample range to identify this subgroup. However, while research by Ling et al. [19] and Picoito et al. [26] have suggested subtypes of internalizing and externalizing problems or age-related distinctive combinations of problems rather than pure problems, other investigators have identified a pure externalizing subgroup with similar age range as the present study (e.g., [79, 80]). A more promising explanation may be the use of distinct informants assessing mental health problems. In contrast to the multi-informant approach (self- and parent-report) in the current study, Duprey et al. [79] and Shi et al. [80] used either parent or teacher reports, respectively. According to Goodman [68], externalizing (conduct and hyperactivity) problems may be more evident depending on the situation and the setting (e.g., home and school). Self-reports may describe worries or fears in terms of socio-emotional problems of teenagers more accurately as hidden from the adults, which may account for the pure internalizing subgroup (i.e., emotional class) in the current sample opposing to the missing internalizing subgroup in Shi et al. [80] and Duprey et al. [79]. Future research should include all possible informants (i.e., self-reports, parents, and teachers) to obtain a clearer and more reliable picture of children and adolescent mental health.

Children and adolescents with externalizing problems seem to prevail within the multiple problem class showing co-occurring mental health problems. Moreover, results indicate that members of the multiple problem class are much younger compared to the members in the other classes. This is in line with previous research suggesting an early onset of co-occurring problems and a persistence into adolescence [16, 26, 56]. However, as expected, a smaller class was extracted characterized by a very high probability for internalizing problems (emotional problem class). In contrast to externalizing problems, internalizing problems are shown to increase due to cognitive maturation during childhood and adolescence [8, 26, 57].

### Stability of class membership over time

The classes replicated at each time point were very similar in terms of structure and proportion with one exception. The emotional problem class showed an increase in the proportion of class membership over time—more individuals were assigned to the emotional problem class in B3 compared to B1. The emotional problem class is characterized by a very high proportion of girls, supporting the large body of evidence outlining the sex differences in the prevalence and trajectories of internalizing problems during childhood and adolescence [10, 57, 81–83]. Consistent gender differences have been documented to emerge during adolescence [84], while not only describing discrepancies in prevalence rates regarding mental health problems but additionally reporting a divergence over time. Internalizing problems have been shown to sharply increase during adolescence (ages 11 to 14) especially for girls [26, 85]. Our results show that although a persisting membership in the emotional problem class was less likely (transition probability 63.2%) compared to the other classes, if stability prevails then females surpass males. In addition, the low persistence of the emotional problem class over time (2.9% of the sample) may be explained by earlier studies suggesting that internalizing problems have a higher tendency to recover by the time of emerging adulthood [86]. A number of explanations for these gender differences have been offered ranging from biological to social influences [87]. For instance, socialisation experiences based on expectation of girls to be preoccupied with interpersonal relationship by being prosocial and submissive compared to boys may explain gender differences for mental health problems [42, 85, 88]. In the present study, a large group of children and adolescents remains stable with low probability of mental health problems (77.3%) similar to previous findings [2]. Future investigations should also focus on the vast majority of children that do not show mental health problems over the lifetime to explore additional protective factors to ensure mental health.

As expected, the multiple problem class showed a high persistence (above 80% transition probability), which is in line with previous research that showed a strong stability of co-occurring social-emotional and behavioural problems for young children [8, 15, 26, 56]. Albeit homotypic stability for the multiple problem class was observed, only an unidirectional heterotypic stability was demonstrated by changing from the multiple problem class to the normative or emotional problem class but not vice versa. According to McElroy et al. [45], there is a moderate chance for children to progress from externalizing to internalizing behaviour, but not reverse. Our findings indicate a very low probability to develop co-occurring externalizing and internalizing problems (i.e., multiple problem class) between B2 and B3, which may be due to the age-dependent decline of externalizing problems. Early childhood seems to be a crucial period for the development of co-occurring problems, which tend to stabilize well into adolescence [57]. The intriguing question of what differentiates individuals with a homotypic stability from individuals with a heterotypic stability of the multiple problem resulting in low level of mental health problems (i.e., normative class) or only internalizing problems remains to be answered by future research. We recommend a closer look at transdiagnostic risk and protective factors for stable and unstable co-occurring mental health problems across childhood and adolescence (e.g., [89]).

### Mental health outcomes and class membership over time

A further aim of the present study was to examine associations between latent class membership for mental health problems and self-reported psychiatric diagnoses (e.g., ADHD, depression, and anxiety). As expected, individuals classified with a mental problem pattern (i.e., emotional problem or multiple problem class) were more likely to report diagnoses of mental disorders (i.e., depression, anxiety, and ADHD).

The stability of the emotional problem class over time was associated with a higher risk for diagnoses of depression and anxiety. In addition, individuals with a stable membership in the multiple problem class analogue to consistent co-occurring externalizing and internalizing problems showed a very high likelihood to report diagnoses of depression, anxiety, and ADHD. This finding is in agreement with existing literature reporting that individuals with co-occurring problems exceed individuals with sole problems regarding their history of mental illness and aggressive behaviour [8, 23]. Moreover, co-occurring problems seem to promote negative peer relationships by which children who are aggressive and depressed are less appreciated by peers compared to being aggressive or depressed [90]. In addition, the stability of membership in the multiple problem class over time is much higher compared to the emotional problem class (11.3% vs. 2.9%), which is in agreement with previous studies that showed a high stability for co-occurring internalizing and externalizing problems [16]. In contrast, individuals with an unstable class membership over time do not show a higher likelihood to report diagnoses of mental disorder compared to individuals with no symptoms over time. One exception is represented by individuals who transition from the multiple problem class to the normative class at time point B3 reporting a diagnosis of ADHD. This finding is not unexpected considering that Attention deficit/hyperactivity disorder is one of the most common mental disorders first detected in childhood [91]. And although ADHD is considered a life-course disorder [92], Biederman et al. [93] state that about 25% with a childhood diagnosis appeared fully remitted (i.e., possible recovery) in adulthood.

### Limitations

Several limitations of the present study are worth mentioning. First, the number of indicators used for the LCA might be considered rather small. However, there is an ongoing discussion about positive impact of fewer indicators as increasing number may lead to data sparseness, low power of chi-square goodness-of-fit tests, and an increase in the number of boundary parameter estimates [94]. Second, dichotomizing continuous SDQ variables into “abnormal” and “normal and borderline” might result in loss of information. Despite this limitation, the present research uses binary data as suggested by Nylund et al. [76] to support interpretation and model accuracy for the LCA. Furthermore, this approach has the advantage that longitudinal categorical shifts between classes can be more definitely identified as persistent cases.

Third, measurement variance across countries regarding the SDQ scores may restrict the generalizability of our findings to other nations [95].

Forth, caution needs to be taken with the interpretation of the transition patterns as sample size is small for each transition group (0.2 – 2.8%) and future studies should aim to replicate results in larger populations. Fifth, the present analysis uses data beginning from middle childhood (age 8) and therefore limits the generalizability of the findings relative to infant children. Future research is needed to explore latent classes of mental health problems in early childhood (preschool years, respectively) as it is characterized as an important developmental stage with the highest sensitivity towards internalizing and externalizing problems [26]. Furthermore, retrospective self-reported diagnoses of mental disorders were used in the analysis which elevates the risk for recall bias. For instance, research by Schlack et al. [96] reported that only 57.4% of parents who had reported a life-time diagnosis of ADHD reported it again after six years. They reasoned that this may be because of changes in relevance of previous diagnoses over years due to child’s recovery or corrected diagnosis. However, the prevalence of ADHD in the current sample of 5.2% was not under- or overestimated compared to the reported worldwide prevalence [97, 98]. Finally, we did not include any treatment variable regarding mental disorders (i.e., depression, anxiety and ADHD) which may have given additional information about remission and change across subgroups over time. Future research should include this aspect.

### Prevention implications

Untreated and co-occurring mental health problems in childhood and adolescence can exert severe and long-term impact on adulthood. The understanding of the stability and manifestation of socio-emotional and behavioural problems is of great importance for the identification of children and adolescents at risk and gives the opportunity to provide early intervention to later diagnoses of mental disorders [19].

Our findings confirm the stability of mental health problems as common rather than atypical.

Fortunately, the majority of children remain stable with low or no mental health problems (77.3%) which supports the salutogenetic approach aimed to further promote and protect health and well-being by strengthening socio-emotional competencies.

Against the finding of a remission rate of 37.7% for mental health problems among individuals with heterotypic stability, 14.7% of individuals in the sample showed a persistent pattern of mental health problems over time, similar to previous findings (e.g., [2]). Targeting those children and adolescents has several implications for prevention and intervention strategies. First, the whole range of mental health problems including co-occurring externalizing and internalizing behaviour should be considered to prevent the risk of manifestation of problems and development of mental disorders later in life [16, 40]. Hence transdiagnostic interventions are imperative and demanded to address both types of problems (i.e., internalizing and externalizing) rather than focusing on one problem [39]. Second, a gender-sensitive approach is essential for diagnosis and treatment of mental health, as strong evidence highlights gender-specific trajectories of mental health problems whereby hyperactivity is more pronounced in boys and anxiety is more often diagnosed for girls [99].

Finally, treatment should be continuous to counter the risk of chronic trajectories of psychopathology. Especially, as the need to address both types of mental health problems will increase the duration of specialized intervention [100, 101]. This claim is in line with meta-analytical evidence linking longer and more intense interventions with better outcomes in different domains such as social-emotional development [102].

## Data Availability

The data that support the findings of this study are available from the Research Devision Child Public health* but restrictions apply to the availability of these data, which were used under license for the current study, and so are not publicly available. Data are however available from the authors upon reasonable request and with permission of the Research Devision Child Public health*. *Research devision Child Public Health, Department of Child and Adolescent Psychiatry, Psychotherapy, and Psychosomatics, University Medical Center Hamburg-Eppendorf, Germany.
